# A comprehensive and cost-effective approach for investigating passive dispersal in minute invertebrates with case studies of phytophagous eriophyid mites

**DOI:** 10.1007/s10493-020-00532-z

**Published:** 2020-08-18

**Authors:** Lechosław Kuczyński, Anna Radwańska, Kamila Karpicka-Ignatowska, Alicja Laska, Mariusz Lewandowski, Brian G. Rector, Agnieszka Majer, Jarosław Raubic, Anna Skoracka

**Affiliations:** 1 Population Ecology Lab, Faculty of Biology, Adam Mickiewicz University, Poznań, Uniwersytetu Poznańskiego 6, 61-614 Poznań, Poland; 2 Section of Applied Entomology, Department of Plant Protection, Institute of Horticultural Sciences, Warsaw University of Life Sciences-SGGW, Nowoursynowska 159, 02-776 Warszawa, Poland; 3 Great Basin Rangelands Research Unit, USDA-ARS, 920 Valley Road, Reno, NV 89512 USA

**Keywords:** Cereal rust mite, Colonization, Dispersal effectiveness, Passive dispersal, Phoresy, Wheat curl mite, Wind dispersal

## Abstract

Dispersal is a fundamental biological process that operates at different temporal and spatial scales with consequences for individual fitness, population dynamics, population genetics, and species distributions. Studying this process is particularly challenging when the focus is on microscopic organisms that disperse passively, whilst controlling neither the transience nor the settlement phase of their movement. In this work we propose a comprehensive approach for studying passive dispersal of microscopic invertebrates and demonstrate it using wind and phoretic vectors. The protocol includes the construction of versatile, modifiable dispersal tunnels as well as a theoretical framework quantifying the movement of species via wind or vectors, and a hierarchical Bayesian approach appropriate to the structure of the dispersal data. The tunnels were used to investigate the three stages of dispersal (viz., departure, transience, and settlement) of two species of minute, phytophagous eriophyid mites *Aceria tosichella* and *Abacarus hystrix*. The proposed devices are inexpensive and easy to construct from readily sourced materials. Possible modifications enable studies of a wide range of mite species and facilitate manipulation of dispersal factors, thus opening a new important area of ecological study for many heretofore understudied species.

**Electronic supplementary material:**

The online version of this article (10.1007/s10493-020-00532-z) contains supplementary material, which is available to authorized users.

## Introduction

Dispersal is a fundamental biological process with consequences for individual fitness, population dynamics, population genetics, and species distributions. Studying dispersal is logistically challenging as this process operates at multiple temporal and spatial scales. It is also a multistage phenomenon, involving departure (initiation of movement), transience (the movement itself) and settlement in a favorable habitat (Clobert et al. 2009, 2012). Simultaneous investigation of all three stages of dispersal is particularly difficult when the focus is on microscopic organisms that disperse passively. Such organisms control neither the transience nor the settlement phase of their dispersal, relying instead on various biotic or abiotic agents, such as wind and water currents or phoretic vectors (Waite and McAlpine 1992; Bell et al. 2005; Reynolds et al. 2006; Galvão et al. 2012; Márquez-Luna et al. 2016; Santos et al. 2020). In spite of the great risk that is inherent to passive dispersal (due to the lack of control over direction and relatively random settlement location), it is a common means of dispersal for minute invertebrates, including mites (Li and Margolies 1993; Parker and Gerson 1994; Sabelis and Bruin 1996; Tixier et al. 1998; Jung and Croft 2001), insects (Moore and Hanks 2004; Smith et al. 2016), spiders, nematodes, tardigrades, collembolans, and rotifers (Ptatscheck et al. 2018). Many species belonging to the aforementioned taxa are integral to ecosystem functions and may also have great invasive potential and economic importance (Hulme 2009; Hoy 2011). Attached to various substrates, they might travel long distances and colonize new regions (Umina et al. 2004) and, in view of climatic changes, may dramatically increase their distributions (Isard and Gage 2001; Travis et al. 2013). Thus, investigation of their spread is critical to studies of species dynamics in populations and communities.

In view of the complex, multi-partite nature of dispersal, tracking the passive movements of minute invertebrates through all stages of dispersal poses great challenges both in the field and under laboratory conditions, due to the technical constraints associated with effectively monitoring virtually invisible subjects (Monfreda et al. 2010). Laboratory-based studies have focused mainly on discrete stages of passive invertebrate dispersal, as such the entire process has rarely been thoroughly analyzed. The settlement phase and the ability to colonize landing sites have been particularly poorly understood, as previous studies have concentrated primarily on departure behaviors (Sabelis and Afman 1994; Osakabe et al. 2008; Melo et al. 2014; Kiedrowicz et al. 2017) or comparisons of different means of dispersal (Yano 2004; Galvão et al. 2012).

Here we propose a comprehensive protocol to accurately measure the passive dispersal of microscopic invertebrates. The protocol includes a theoretical framework quantifying the movement of invertebrate species that disperse passively via wind or via phoretic vectors, equipment construction, and a hierarchical Bayesian approach to estimate dispersal parameters. Our approach provides a useful new tool for studies pertaining to the different phases of dispersal, viz., departure, transience, and settlement. To illustrate the utility of our devices and protocol, we present case studies of two phytophagous eriophyid mite species with different dispersal agents and host plants. Adoption of our protocol should accelerate research progress in the field of dispersal ecology of tiny, passively spreading invertebrates and lead to a better understanding of how and why these organisms move and the larger implications of these overlooked movements.

## Methods

### Equipment

The device consists of several basic elements: an axial fan with a power regulator to generate wind of a desired velocity, a flow straightener (to reduce stream turbulence), a transparent tube (forming the departure and transience space) and a target module (containing a habitat patch suitable for settlement and colonization). This general design can be modified according to the phase or mode of dispersal or cue being tested.

### ‘Wind-transience’ tunnel

**Fig. 1:**
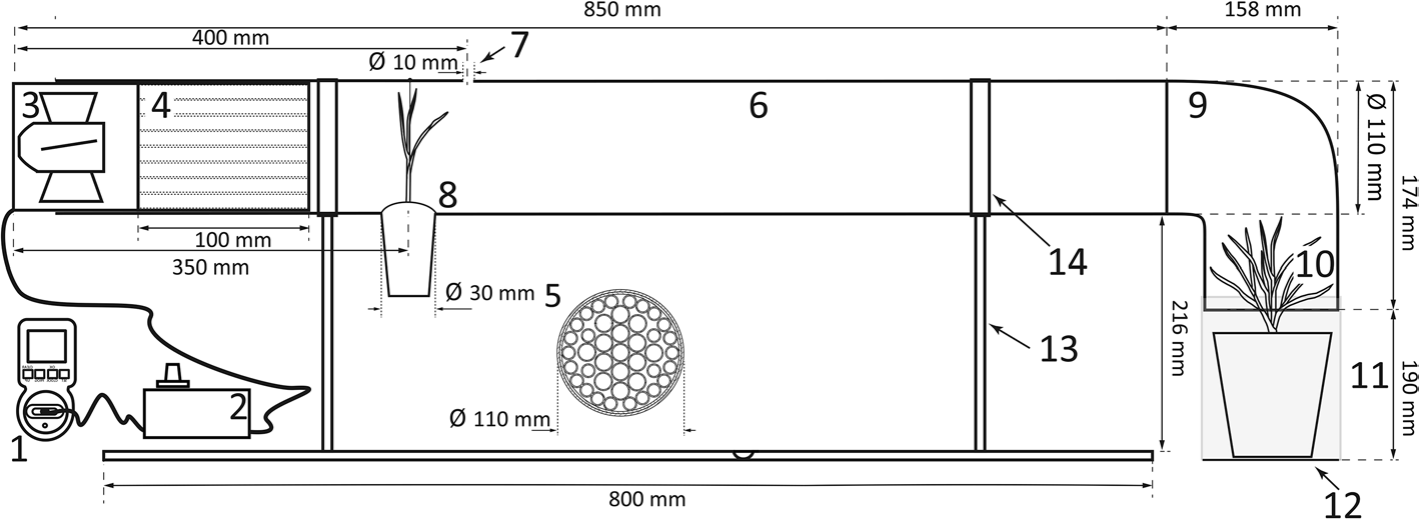
Scheme of a ‘wind-transience’ tunnel: (1) timer, (2) power regulator to control wind speed, (3) axial fan, (4) flow straightener consisting of small tubes that form (5) honeycomb-like structure in cross-section, (6) transparent tube, (7) hole for anemometer, (8) source patch (for plants a hole was cut at the bottom to seat the pot), (9) elbow connector, (10) target module, (11) polyamide sleeve to protect the target patch, (12) platform, (13) brackets, (14) clamps. For technical drawing, photo and video see Online Resource 1 (ESM_1.pdf) and Online Resource 5 (ESM_5.mp4): https://doi.org/10.5281/zenodo.3964151

The following arrangement allows the assessment of the departure, the transience stage of dispersal via wind currents and subsequent settlement on a target patch. At the windward end of the transparent tube there is a source patch from which invertebrates disperse. A hole cut in the top accommodates an anemometer. An elbow connector fitted to the distal end is attached to a target module on which dispersing invertebrates would settle. The target patch is protected with a polyamide sleeve against contamination from outside the experimental space (Fig. 1; Online Resource 1 (ESM_1.pdf): Figs S1, S4; Online Resource 5 (ESM_5.mp4): https://doi.org/10.5281/zenodo.3964151). This device allows for long-term testing of the transience phase during which both wind speed and wind regimes are regulated using a programmable electronic timer.

### ‘Vector-transience’ tunnel

**Fig. 2:**
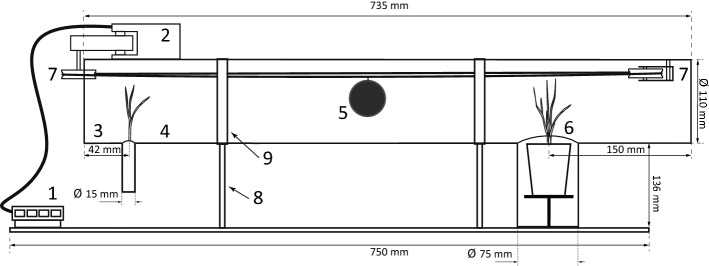
Scheme of a ‘vector-transience’ tunnel: (1) control panel, (2) motor, (3) transparent tube, (4) source patch, (5) an artificial vector (a ping-pong ball ⌀ 40 mm covered with wool to imitate a mammalian vector), (6) target patch, (7) two pulleys, (8) brackets, (9) clamps. For technical drawing, photo and video see Online Resource 1 (ESM_1.pdf) and Online Resource 6 (ESM_6.mp4): https://doi.org/10.5281/zenodo.3964151

The vector-transience model enables testing of the departure and the transience phase using a vector, followed by settlement of study subjects on a target patch. In place of wind-generating elements, both ends of the tube are closed and a vector is introduced inside the tube. Living invertebrates (e.g. insects) can be inserted together with the source patch and allowed to move between source and target patches. Mammalian vectors can be imitated using a simple robot. Robotic mammalian vector’s movement can be effectuated using Lego Mindstorms NXT 2.0 or a similar mechanical system. Atop the tube at either end, two pulleys are installed with a programmable motor attached to the one of them; flexible string is looped between them to convey the attached vector between the two patches (Fig. 2; Online Resource 1 (ESM_1.pdf): Figs S2, S5; Online Resource 6 (ESM_6.mp4): https://doi.org/10.5281/zenodo.3964151).

### Example of possible modification: ‘departure’ tunnel

**Fig. 3:**
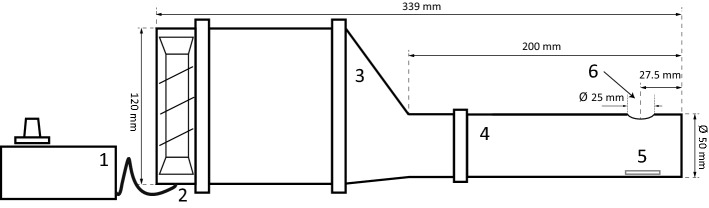
Scheme of a ‘departure’ tunnel: (1) power regulator to control wind speed, (2) axial fan, (3) convergent pipe, (4) transparent tube, (5) experimental arena, (6) hole enabling stereomicroscopic observations and wind speed measurements*.* For technical drawing, photo and video see Online Resource 1 (ESM_1.pdf) and Online Resource 7 (ESM_7.mp4): https://doi.org/10.5281/zenodo.3964151

The above described equipment can be modified in various ways. Below, we show a detailed description of such a modification, in which a transparent tube was fitted to a stereomicroscope (with a digital camera connected to a computer) to directly record departure events and dispersal related behaviors in the presence of wind. At the open end of the tube there is a hole cut at the top which enables wind speed measurement using an anemometer and stereomicroscopic observations of mite behavior in the experimental arena, which is directly below the hole (Fig. 3; Online Resource 1 (ESM_1.pdf): Figs S3, S6; Online Resource 7 (ESM_7.mp4): https://doi.org/10.5281/zenodo.3964151).

### Quantifying dispersal

**Fig. 4:**
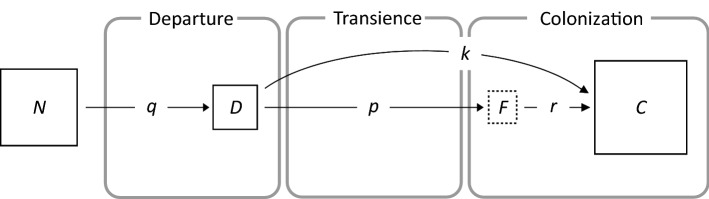
Approach for estimating quantities that measure dispersal. Boxes represent sets of individuals of a size denoted by the uppercase letters at different stages of dispersal (rounded boxes): *N*—population size on the source patch; *D*—no. of dispersers, i.e., individuals that leave the source patch; *F*—no. of founders, i.e., individuals that successfully arrive and settle on the target habitat patch; *C*—no. of colonizers, i.e., population size on the target patch after several generations. Solid boxes are numbers that can be observed and the dashed box is an unobservable quantity (latent variable). Arrows represent processes and lowercase letters are measures that quantify those processes

Our approach allows elucidation of the multi-stage characteristics of dispersal and to assess each stage quantitatively using data collected by the above described tunnels. To quantify dispersal on the basis of the data collected using the equipment described above, we used the following measurements (see Fig. 4 for explanation of symbols):

1. Dispersal rate: proportion of dispersers in a population, i.e., the no. of dispersers to the no. of all individuals in the source population (*q* = *D*/*N*).
2. Dispersal effectiveness: proportion of successful dispersers, i.e., the no. of founders to the no. of dispersers (*p* = *F*/*D*).
3. Population growth rate: intrinsic rate of population increase, expressed as *r* = log_2_(*C*/*F* + 1). This formulation allows for a straightforward and meaningful interpretation: if *r* = 1, the population size did not change; if *r* > 1 the population increased; if *r* < 1 the population decreased. If the entire population of colonizers goes extinct, *r* = 0.
4. Colonization potential: proportion of colonizers to dispersers (*k* = *C*/*D* or, equivalently: *k* = *p*(2^*r*^-1)). This measure combines dispersal effectiveness and population growth on a newly colonized patch.

In our study system (which is analogous to other systems with tiny propagules), counting individuals immediately after they land on a target habitat patch (a plant in our case) was technically not feasible because of the risk of damaging the plants while searching for scattered dispersers within plant structures. In addition, recently landed mites could be tossed from the leaves during inspection. Thus, we did not attempt to estimate the number of mites that successfully landed, rather allowing them to multiply for several generations and estimating population size thereafter. By knowing population growth rate at a specific rearing temperature (Kuczyński et al. 2016), we were able to infer the number of individuals that successfully landed and multiplied on the target plant.

To do so, we used a Bayesian hierarchical modelling approach (see Online Resource 2–3 (ESM_2.R and ESM_3.R) for the specification of the model in the JAGS language: https://doi.org/10.5281/zenodo.3964151). We assumed that during each experimental trial $i$, the number of individuals on the target plant two generations after settlement follows the Poisson distribution with the mean ${\lambda }_{i}$:

$$C_{i} \sim Poisson\left( {\lambda_{i} } \right).$$

The value of ${\lambda }_{i}$ is the expected number of individuals given that the population growth rate is $r$ and the initial no. of individuals (i.e., the no. of founders) was ${F}_{i}$. Recalling our definition of population growth rate (above), we get ${\lambda }_{i}={F}_{i}({2}^{r}-1)$. The population growth rate $r$ was estimated based on our previous work (Kuczyński et al. 2016) and entered into the model as an informative prior:

$$r\sim Norm\left( {m,\sigma } \right),$$

where both parameters of the distribution of $r$ (i.e., the mean $m$ and the standard deviation $\sigma$) were calculated from the model describing the functional relationship between the temperature and population growth rate, estimated under controlled laboratory conditions (Fig. 2 in Kuczyński et al. 2016). The mean $m$ was calculated as the predicted value of the population growth rate at 27 °C (used in our experiments for incubating mites after the settlement) and the standard deviation $\sigma$ was estimated by using a second-order Taylor expansion and Monte Carlo simulation (function ‘predictNLS’ implemented in the R package ‘propagate’ (Spiess 2018)).

The number of founders ${F}_{i}$ was an unobservable (latent) variable due to technical constraints mentioned above. It was assumed to follow the binomial distribution with an index parameter ${D}_{i}$, representing the number of dispersers and a success parameter ${p}_{i}$, representing dispersal effectiveness:

$$F_{i} \sim Binom\left( {D_{i} ,p_{i} } \right).$$

To account for additional variation in dispersal effectiveness not captured by the binomial distribution itself (i.e., overdispersion), a normally distributed random effect ${\eta }_{i}$ was added to the linear predictor:

$$logit\left( {p_{i} } \right) = \eta_{i} ,$$

$$\eta_{i} \sim Norm\left( {\mu ,\sigma_{p} } \right),$$

where $\mu$ is a logit-transformed parameter for expected probability of dispersal effectiveness and ${\sigma }_{p}$ is the standard deviation of a random intercept, which is estimated from the data.

Finally, the number of dispersers ${D}_{i}$ was modeled as a binomially distributed variable with parameters ${N}_{i}$ and ${q}_{i}$, representing the number of individuals on the source plant and dispersal rate, respectively:

$$D_{i} \sim Binom\left( {N_{i} ,q_{i} } \right).$$

### Case studies

#### Study system

We demonstrated the use of our instrumentation and approach on two phytophagous eriophyid mites, which are among the smallest of all arthropods (< 300 µm long): wheat curl mite, *Aceria tosichella* (WCM hereafter), and cereal rust mite, *Abacarus hystrix* (CRM hereafter). They are known to disperse passively via wind currents or vectors, infest a wide range of grass species (both cultivated and wild) and cause important economic damage to cereal crops (Frost and Ridland 1996; Sabelis and Bruin 1996; Navia et al. 2010). Both species also represent complexes of cryptic species, consisting of multiple genetically divergent lineages differing in their host plant ranges (Skoracka et al. 2018a; Laska et al. 2018). In this study we worked with the CRM complex 2 associated with wheat (*Triticum aestivum*) and quackgrass (*Elymus repens*) (Laska et al. 2018), and with the most globally distributed WCM lineage (herein MT-1, also known as type 2) associated with cereals (Skoracka et al. 2014, 2017, 2018b). Experimental individuals of both species were obtained from laboratory stock colonies reared (each species separately) for several years on wheat in the laboratory of the Faculty of Biology, Adam Mickiewicz University, Poznań, Poland.

#### Testing the dispersal of WCM in ‘wind-transience’ tunnel

To demonstrate the utility of our approach, we performed two experiments, testing WCM wind dispersal towards wheat (denoted as a case study 1) and smooth brome (*Bromopsis inermis*; case study 2). These two host plant species are known to differ in their suitability as hosts for WCM (Skoracka et al. 2013). In both experiments, the source patches consisted of a single mite-infested wheat plant, and the target patches consisted of 10 clean plants, either wheat or smooth brome. Wheat plants were transplanted directly from the stock colony and mites in the source population were counted (this gives the estimate of *N*—see ‘Quantifying dispersal’ above). The experiments (in 10 replicates for wheat and eight replicates for smooth brome) ran for 24 h at 2.5 m/s wind speed, under regimes that were intended to mimic variable natural conditions in which the wind blows intermittently. These regimes were (minutes blowing/minutes not blowing; the blowing time is in boldface): **5**/55; **15**/45; **30**/30; **60**/60; **120**/120; **180**/180; **180**/180; **180**. Altogether, the wind blew during 770 min (12 h, 50 min), whereas the windless conditions lasted 670 min (11 h, 10 min). After 24 h, mites remaining on the source plant were counted again and this number was subtracted from *N* to estimate the number of dispersers *D*. Target plants (possibly with founder individuals) were incubated in 27 °C, 16:8 D/N, 60% RH for 14 days (ca. two generations), and afterwards mite individuals were counted (giving the estimate of *C*).

#### Testing the dispersal of WCM in ‘vector-transience’ tunnel

In this study (which we denote as a case study 3) a paired set of source (infested) and target (clean) wheat plants constituted an experimental unit. The general experimental setup was the same as in case studies 1 and 2, but with an important modification: instead of wind, an imitation biotic vector was used that moved through the middle of the tube toward the source patch, made contact with the plant, then moved in the opposite direction to make contact with the target patch. The motor was programmed to imitate a simplified scheme of natural activity of mammals that coexist with mites in the field (Online Resource 1 (ESM_1.pdf) Fig. S7: https://doi.org/10.5281/zenodo.3964151). The experiment was replicated nine times and the procedure of counting mites was identical as in the previously described experiments.

#### Testing WCM dispersal rate from two plant species

In this case study (no. 4) the arena consisted of leaf fragments supporting mite specimens placed on agar blocks prepared from modified Murashige and Skoog (1962) (MS) medium (Karpicka-Ignatowska et al. 2019). We used two plant species: wheat and smooth brome. Ten to 17 WCM females were transferred under the stereomicroscope from the stock colony to leaf fragments of each plant species and allowed 30 min to acclimate to the new environment (in 10 replicates for wheat and 11 replicates for smooth brome). After the acclimatization period, mites were counted (giving the estimate of *N*), and an agar block supporting a leaf fragment with mites infested on it was placed inside the departure tunnel for a five-minute blowing session, during which the mites’ behaviors and departure events were recorded using a Canon Mark II 7D video camera. Wind was generated with a stable speed of 3.7 m/s, which was measured using a Testo 405i anemometer. Afterwards, videos were analyzed and all departure events were counted (this gives the estimate of *D*). Dispersal rates for each host plant species were estimated using a GLM model with a binomial distribution for the response and the logit-link function. Differences in dispersal rates between groups (host plant species) were tested using a Likelihood-ratio test.

#### Testing WCM and CRM dispersal rate

The general principle of how this experiment (case study 5) was conducted was the same as in case study 4; however, the following experimental conditions were modified: (a) departure of two eriophyid mite species was quantified, namely WCM and CRM, which were reared altogether in a mixed colony for at least three generations before the experiment, and (b) 20–21 females of each species were separately transferred to wheat leaf fragments in 20 replicates per mite species. Statistical analysis followed case study 4.

## Results

Our simple, cost-effective and flexible set of tools allowed comprehensive testing of all stages of passive dispersal of a tiny phytophagous mite. The ‘wind-transience’ tunnel cost ca. $133, the ‘vector-transience’ tunnel cost ca. $357, and the ‘departure’ tunnel cost ca. $51 (Online Resource 4 (ESM_4.pdf): https://doi.org/10.5281/zenodo.3964151).

The ‘wind-transience’ tunnel (case studies 1 and 2) and vector-transience’ tunnel (case study 3) allowed effective estimation of all parameters quantifying dispersal (and their uncertainty) (Table 1) using the proposed hierarchical Bayesian approach and taking into account the hidden nature of some stages (unobservable states) in a way that reflects the structure of the dispersal process itself (Fig. 4, see Online Resource 2–3 (ESM_2.R and ESM_3.R) for reproducible code in R on how to estimate dispersal parameters: https://doi.org/10.5281/zenodo.3964151).

**Table 1:** Posterior means and 95% credible intervals for dispersal parameters estimated using the ‘wind-transience’ and ‘vector-transience’ tunnels and the wheat curl mite as a study system (results of case studies 1–3)

Parameter	Meaning	Wind to wheat			Wind to brome			Vector to wheat		
		Posterior mean	2.5% quantile	97.5% quantile	Posterior mean	2.5% quantile	97.5% quantile	Posterior mean	2.5% quantile	97.5% quantile
$q$	Dispersal rate	0.366	0.359	0.373	0.127	0.122	0.132	0.575	0.560	0.591
$p$	Dispersal effectiveness	0.286	0.093	0.614	0.008	0.002	0.018	0.009	0.000	0.029
$r$	Population growth rate	3.976	3.665	4.525	1.321	0.742	1.860	4.186	3.493	4.873
$k$	Colonization potential	4.813	4.584	5.037	0.012	0.005	0.021	0.310	0.158	0.874
${\sigma }_{p}$	Standard deviation of the random intercept	1.814	1.071	3.165	0.610	0.031	1.689	1.918	0.615	5.012

**Fig. 5:**
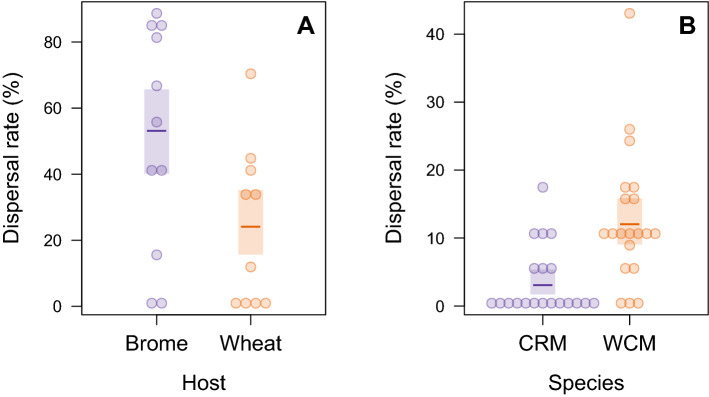
Comparison of dispersal rates of: **a** wheat curl mite (WCM) from smooth brome and wheat, and **b** cereal rust mite (CRM) and WCM from wheat. Points are the observed values (i.e., percentages of dispersers in a population) and short horizontal lines represent estimated means. Shaded bars represent 95% confidence intervals around these means

The ‘departure’ tunnel allowed for documentation of mite behaviors when they were exposed to wind (Online Resource 8 (ESM_8.mp4): https://doi.org/10.5281/zenodo.3964151). Using this device we estimated the dispersal rates of WCM from two host plants (case study 4), which differed significantly (LR test, χ^2^ = 24.0, df = 1, p < 0.001) and were almost twice as high from smooth brome (53.1%, 95% confidence interval [95% CI]: 40.1–65.7) than from wheat (24.1%, 95% CI: 15.6–35.2) (Fig. 5a). The comparison of dispersal rates of two eriophyid species (case study 5) also showed significant differences (LR test, χ^2^ = 13.2, df = 1, p = 0.003): WCM disperses 4 × more frequently (12.0%, 95% CI: 9.1–15.8%) than CRM (3.0%, CI: 1.7–5.4%) (Fig. 5b).

## Discussion

For minute invertebrates, passive dispersal has been poorly studied compared to active movement (Clobert et al. 2009; Bonte et al. 2012), typically in discrete, separate phases (Bonte et al. 2012), hindering comprehensive investigation of this fundamental ecological function. Here, we report studies of minute phytophagous mite species that demonstrate the utility of a novel comprehensive protocol for studying all phases of invertebrate passive dispersal, including instrument construction, a theoretical framework for quantifying dispersal, and a hierarchical Bayesian approach to analysis. As illustrated by the several case studies presented here this versatile device and analytical approach combine to provide a powerful tool to examine the multistage dispersal process of the microscopic mites.

In the case studies 1 and 3, we demonstrated that although dispersal rate (reflecting the departure stage of dispersal) was higher when the phytophagous wheat curl mite (WCM) dispersed via a phoretic vector, the dispersal effectiveness and colonization potential (that result from transience and settlement dispersal stages) were higher when mites moved on wind currents (Table 1). This outcome, revealing that departure rate was not directly correlated with successful settlement in a new habitat, raises questions regarding the costs of dispersal and its associated trade-offs, which are ubiquitous the among different phases of dispersal (Bonte et al. 2012). Using the ‘wind-transience’ tunnel, we also showed (case studies 1 and 2) that WCM individuals dispersed less readily and frequently when smooth brome was their target plant compared to wheat, and consequently the dispersal effectiveness and colonization potential were lower on smooth brome than on wheat (Table 1). Additionally, direct observation of WCM dispersal rate from smooth brome and wheat using our ‘departure’ tunnel (case study 4) supported the above findings (Fig. 5a). Conclusions from case studies 1, 2 and 4 are also in accordance with our previous results showing that WCM host plants differ in their ability to support WCM population growth (Skoracka et al. 2013) and suggest that dispersal is affected by habitat quality.

As the ‘departure’ tunnel also allowed microscopic observation of behaviors that mite individuals expressed in the presence of a dispersal cue viz., wind (Online Resource 8 (ESM_8.mp4): https://doi.org/10.5281/zenodo.3964151), further study may identify specific phenotypic traits associated with dispersal and explain the differences between dispersers and residents. For example, this tunnel has been successfully applied to the question of whether departure events in WCM are phenotype- and context-dependent in relation to behavior, morphology, and environment (Laska et al. 2019). Those authors found that a behavior consisting of forming chains among mites increased the probability of dispersal via wind and that individual mites dispersing in chains were more elongated. In another study, this ‘departure’ tunnel was used to test whether in the presence of hypothetical dispersal cues (e.g. wind), an insect vector and a fresh plant, WCM and CRM display behaviors indicating their readiness to depart from their current plant (Kiedrowicz et al. 2017). These authors demonstrated that both eriophyid mite species only slightly responded to potential dispersal cues, however there were significant behavioral differences between WCM and CRM reflected by higher general activity of WCM. This latter outcome is in concordance with the result of our case study 5, which showed that CRM initiated dispersal significantly less often than WCM (Fig. 5B). It has been already previously shown that high behavioral activity can be positively correlated with high dispersal propensity (Cote et al. 2010; Chapple et al. 2012).

All these examples confirm the utility of described equipment and approach, examining various ecological questions under a diverse array of biotic and abiotic conditions offering insights into mechanistic determinants of dispersal and associated constraints, and expanding our ability to study dispersal syndromes (Ronce and Clobert 2013).

Our approach can also be applied to experimental selection for and against dispersal propensity and dispersal ability by selecting the ‘best’ dispersers (i.e., the individuals that reach the target patch) and ‘worst’ (those that do not leave the source patch) by subjecting them to repeated dispersal sessions through multiple generations. Such method that follows real-time evolution of populations represent a powerful tool to address important ecological and evolutionary phenomena (Kawecki et al. 2012; Magalhães and Matos 2012).

While we described the application of the dispersal tunnels and the analytical framework to the phytophagous mite species that we maintain in our laboratory, our approach should be modifiable and applicable to other minute, passively dispersing organisms, whether phytophagous or not; in the latter case plants could be replaced by an appropriate substrate (e.g. a customized diet). Technical parameters of the device, including the length of the tunnel, the power of the wind, the blowing regime or vector-robot programming can be readily modified to suit a given study system.

In summary, the dispersal tunnels and associated analytical approach presented here are comprehensive and flexible tools to study all stages of passive dispersal (departure, transience and successful settlement) in mites that are invisible to naked eye. The advantages of our approach are based on simplicity and low cost, modification opportunities that should encourage investigation of dispersal in other minute species, the ability to examine the effects of different factors on dispersal, and the execution of selection experiments. In this way, we are opening a microscopic, understudied, but exciting world to a broad spectrum of ecological study. This innovation will accelerate and improve investigation of passive dispersal, allowing a more complex examination of diverse ecological and evolutionary hypotheses leading to a better understanding of the mechanisms and consequences of passive dispersal.

## Electronic supplementary material

Below is the link to the electronic supplementary material.

Supplementary file1 (PDF 792 kb)

Supplementary file2 (R 3 kb)

Supplementary file3 (R 2 kb)

Supplementary file4 (PDF 166 kb)

Supplementary file5 (MP4 4364 kb)

Supplementary file6 (MP4 2776 kb)

Supplementary file7 (MP4 1210 kb)

Supplementary file8 (MP4 17387 kb)

## Data Availability

The datasets generated and analyzed during the current study are available in the Zenodo repository under: https://doi.org/10.5281/zenodo.3963147. Electronic Supplementary Material for the current study is available in the Zenodo repository under: https://doi.org/10.5281/zenodo.3964151.

## Code availability

Custom code used in this study is available in the Zenodo repository under: https://doi.org/10.5281/zenodo.3964151.
