# Supplementary material for: A comprehensive and cost-effective approach for investigating passive dispersal in minute invertebrates with case studies of phytophagous eriophyid mites
Source: Exp Appl Acarol. 2020 Aug 18;82(1):17–31. doi: 10.1007/s10493-020-00532-z (PMC7471196; doi:10.1007/s10493-020-00532-z)
Supplement: Supplementary file 1 — Supplementary file1 (PDF 792 kb) [file 10493_2020_532_MOESM1_ESM.pdf]

Online Resource 1

A comprehensive and cost-effective approach for investigating passive dispersal in minute invertebrates with case studies of phytophagous eriophyid mites

Lechosław Kuczyński, Anna Radwańska, Kamila Karpicka-Ignatowska*, Alicja Laska, Mariusz Lewandowski, Brian G. Rector, Agnieszka Majer, Jarosław Raubic, Anna Skoracka

*corresponding author

Table of contents

Figure S1 Photo of the 'wind-transience' tunnel used to test the transience stage with wind currents and to test subsequent settlement on the target patch.. 2
Figure S2 Photo of the 'vector-transience' tunnel used to test transience phase with vector and subsequent settlement. 3
Figure S3 Photo of the 'departure' tunnel used for direct observation and recording of departure events and dispersal-related behaviors in the presence of wind 4
Figure S4 Technical drawing of the 'wind-transience' tunnel. 5
Figure S5 Technical drawing of the 'vector-transience' tunnel. 6
Figure S6 Technical drawing of the 'departure' tunnel. 7
Figure S7 The scheme of Lego Mindstorms NXT 2.0 – engine program for a robotic mammalian vector simulation. 8

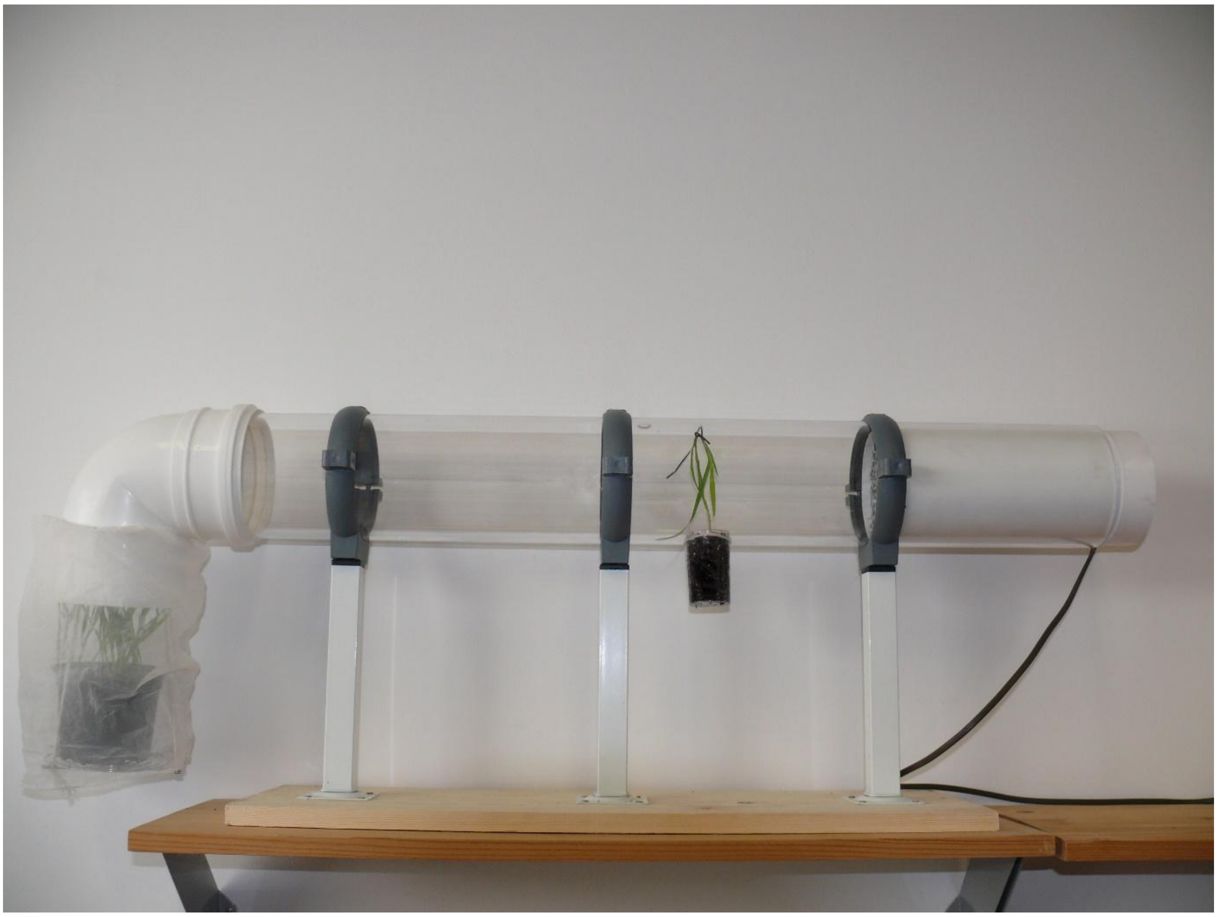

Fig. S1 Photo of the ‘wind-transience’ tunnel used to test the transience stage with wind currents and to test subsequent settlement on the target patch.

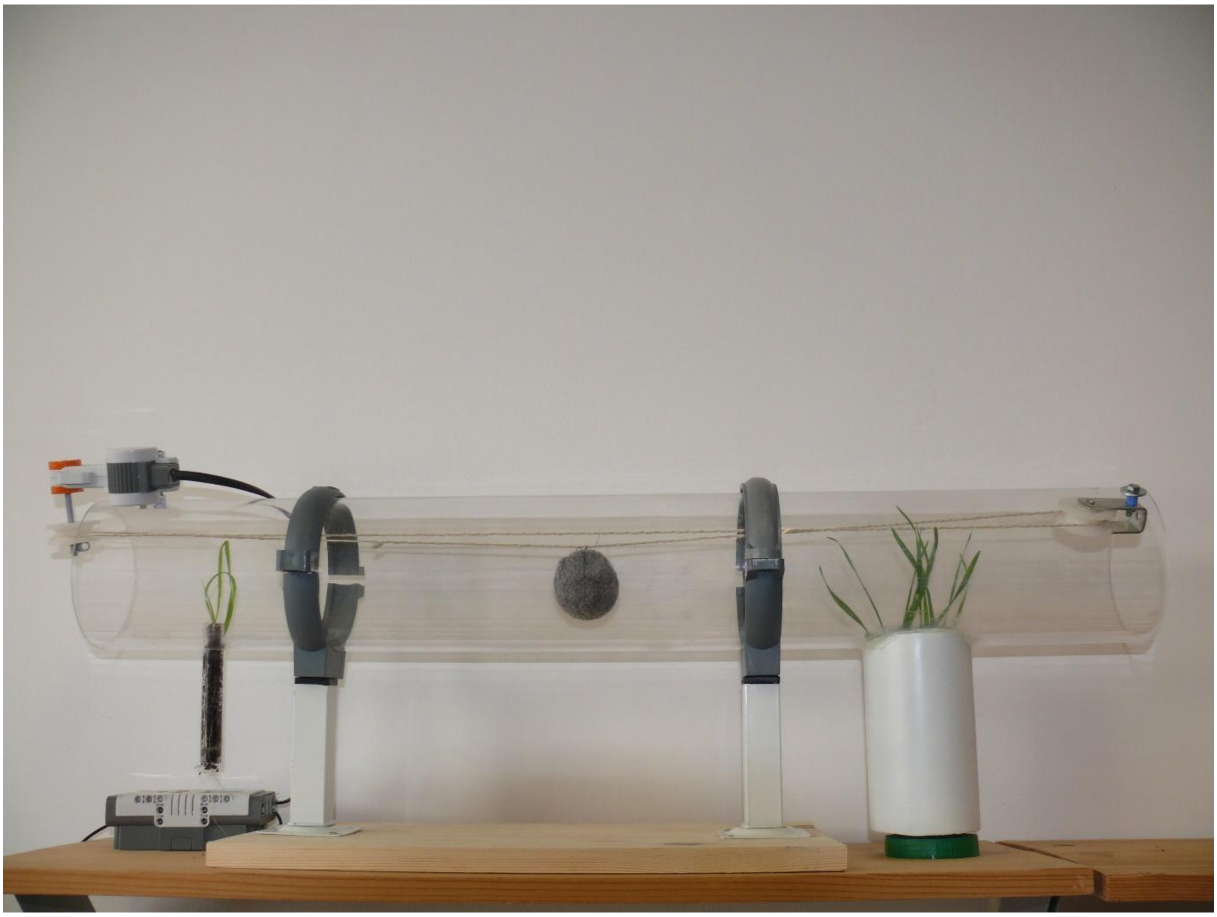

Fig. S2 Photo of the ‘vector-transience’ tunnel used to test transience phase with vector and subsequent settlement.

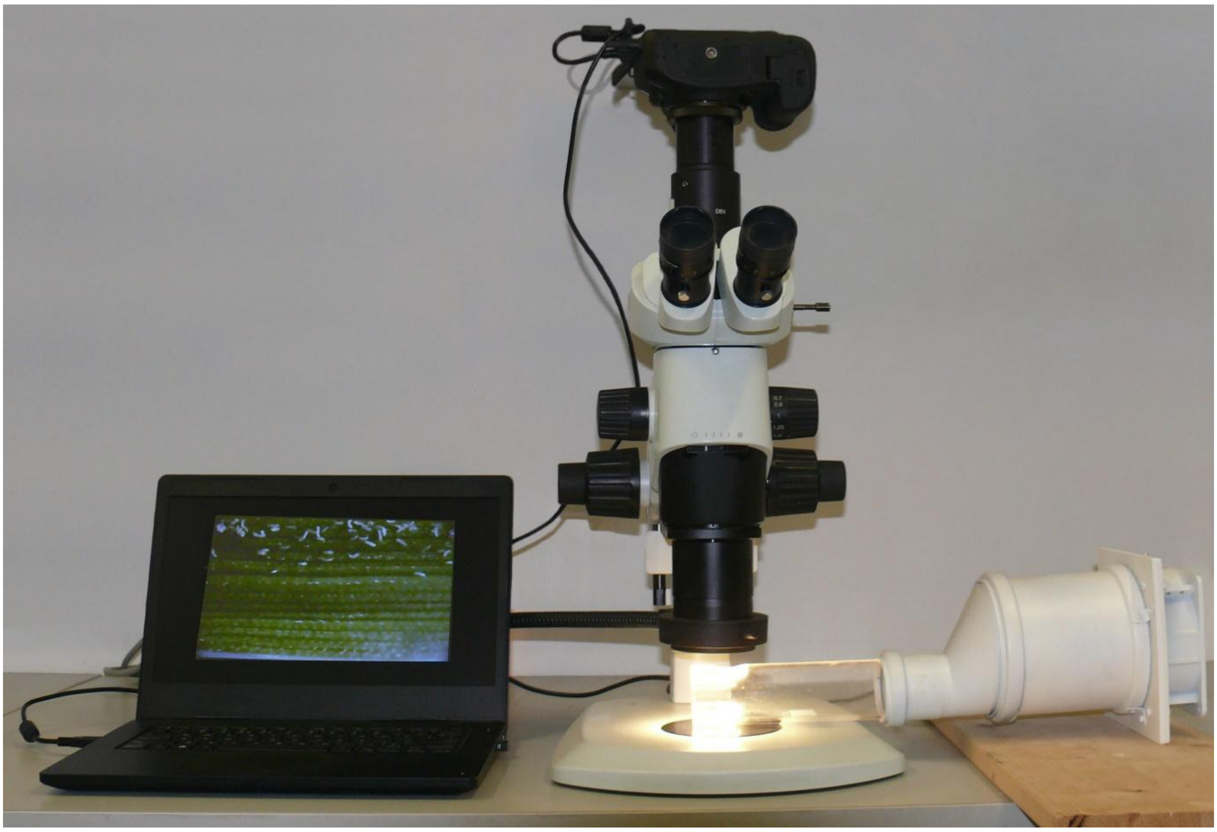

Fig. S3 Photo of the ‘departure’ tunnel used for direct observation and recording of departure events and dispersal-related behaviors in the presence of wind.

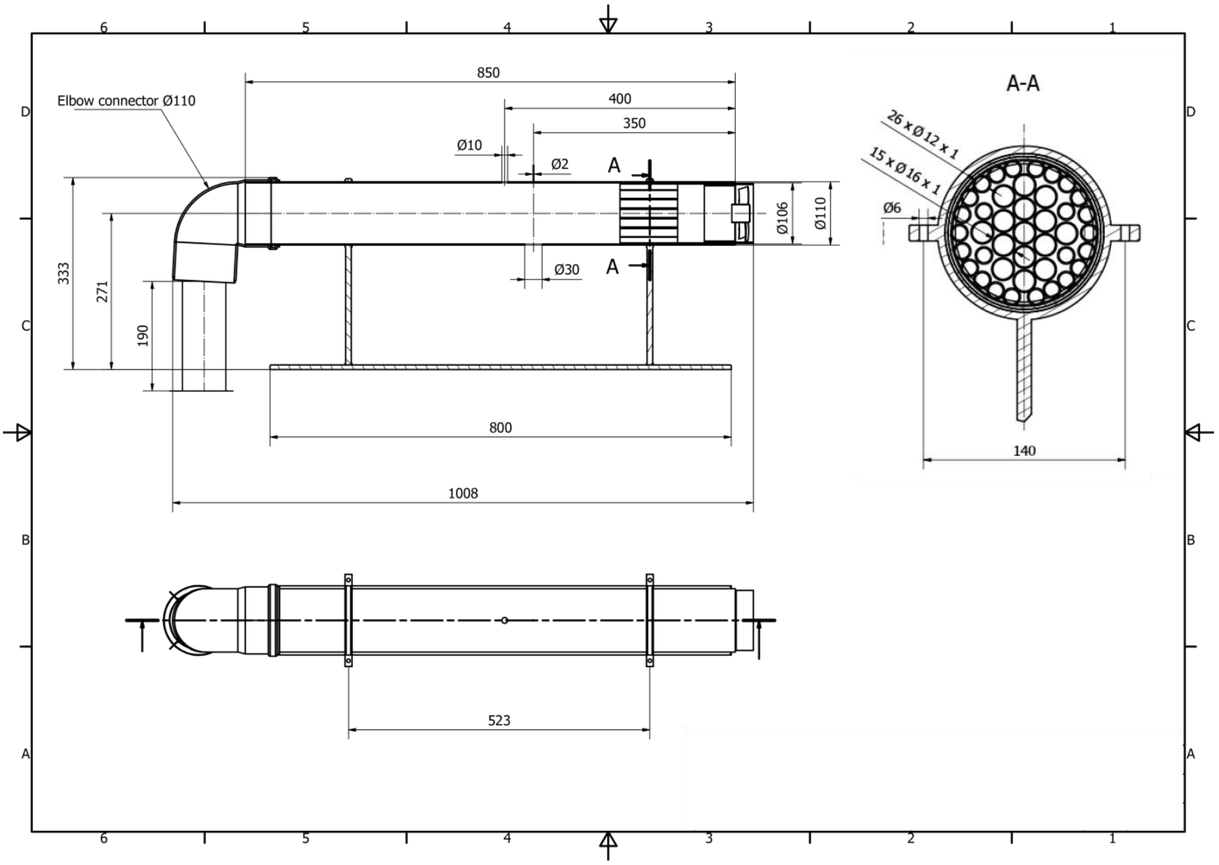

Fig. S4 Technical drawing of the 'wind-transience' tunnel. All measurements are given in millimeters.

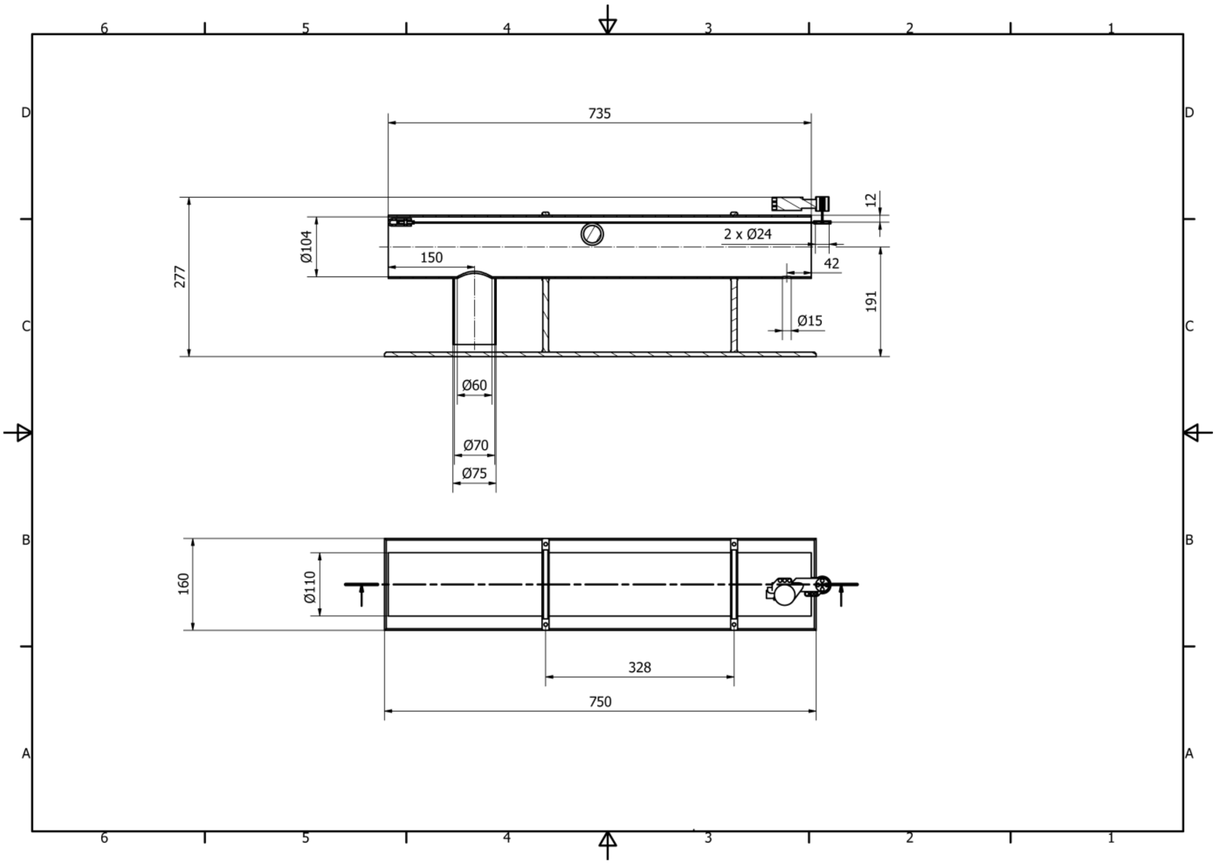

Fig. S5 Technical drawing of the 'vector-transience' tunnel. All measurements are given in millimeters.

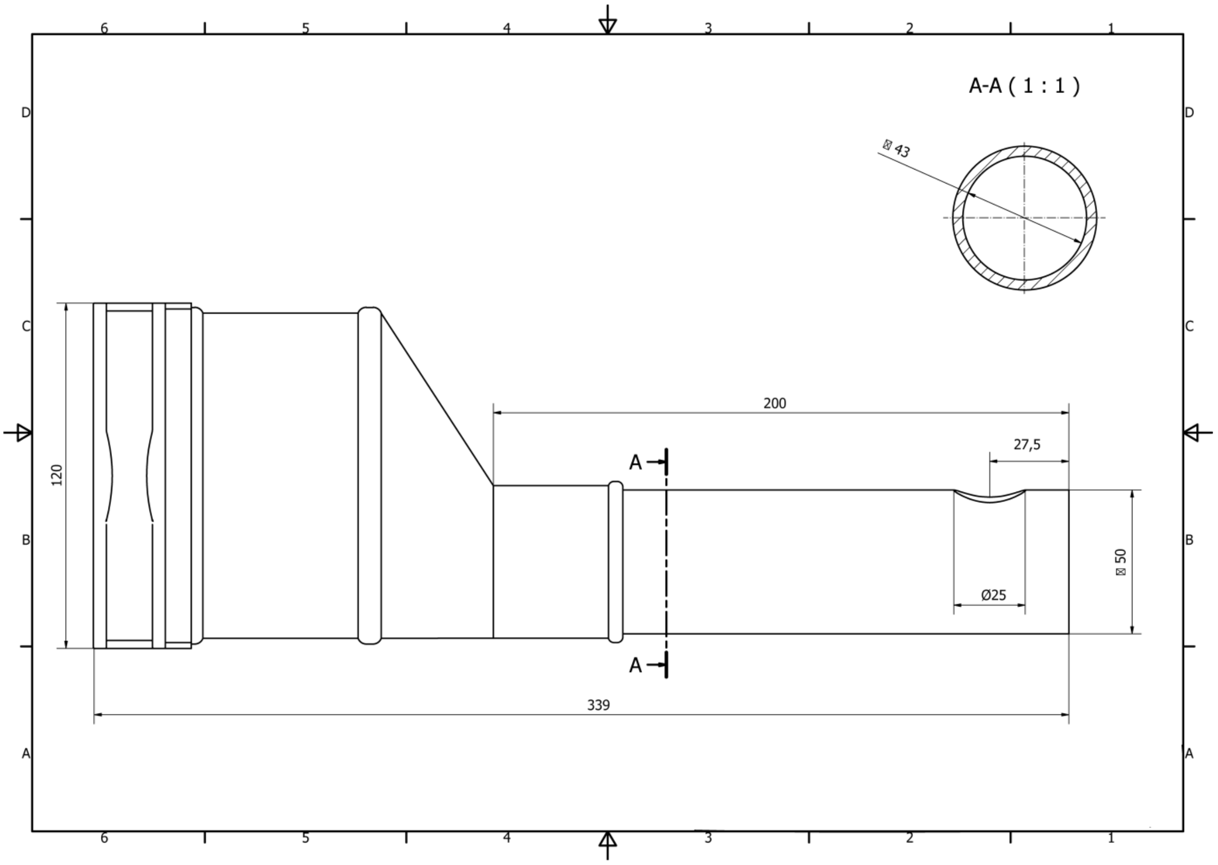

Fig. S6 Technical drawing of the 'departure' tunnel. All measurements are given in millimeters.

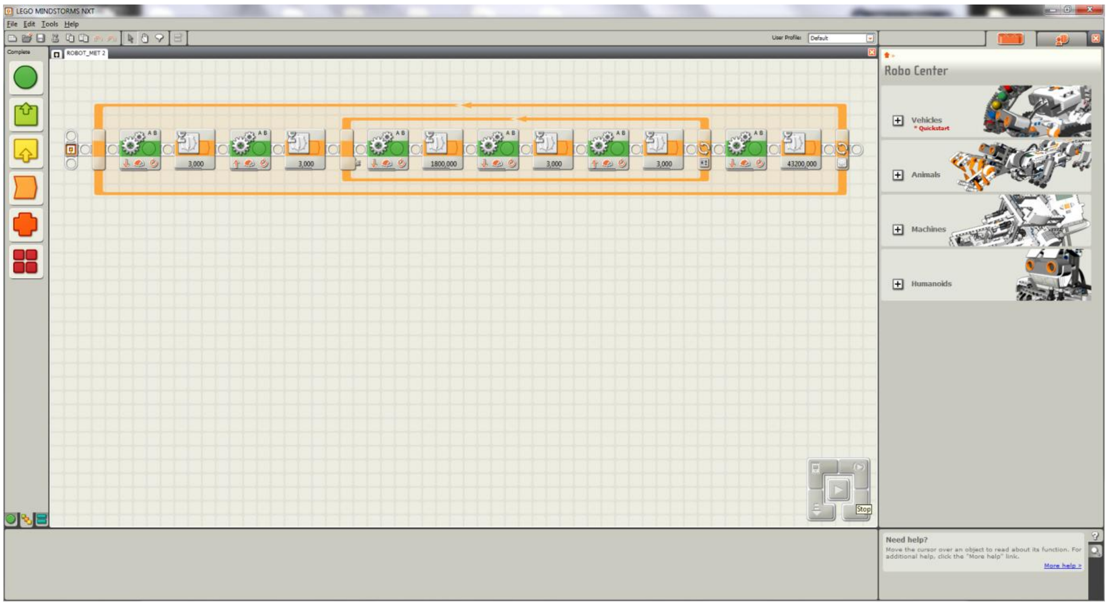

Fig. S7 The scheme of Lego Mindstorms NXT 2.0 – engine program for a robotic mammalian vector simulation.

Action/flow blocks:

1. Movement from the middle of the tube to the source plant
2. 3-second stop over the source plant
3. Movement from the source plant to the target plant
4. 3-second stop over the target plant
5. Movement from the target plant to the middle of the tube
6. 30-minute stop in the middle of the tube
7. Movement from the middle of the tube to the source plant
8. 3-second stop over the source plant
9. Movement from the source plant to the target plant
10. 3-second stop over the target plant
11. Movement from the target plant to the middle of the tube
12. 12-hour stop in the middle of the tube

Small loop – the cycle repeated 12 times (points: 5 – 10)

Big loop – the cycle repeated 2 times (points: 1 – 12)

In the beginning of the second 12-hour stop, the entire robot was shut down and the target plants were incubated.
