# Supplementary material for: A comprehensive and cost-effective approach for investigating passive dispersal in minute invertebrates with case studies of phytophagous eriophyid mites
Source: Exp Appl Acarol. 2020 Aug 18;82(1):17–31. doi: 10.1007/s10493-020-00532-z (PMC7471196; doi:10.1007/s10493-020-00532-z)
Supplement: Supplementary file 4 — Supplementary file4 (PDF 166 kb) [file 10493_2020_532_MOESM4_ESM.pdf]

Online Resource 4

A comprehensive and cost-effective approach for investigating passive dispersal in minute invertebrates with case studies of phytophagous eriophyid mites

Lechosław Kuczyński, Anna Radwańska, Kamila Karpicka-Ignatowska*, Alicja Laska, Mariusz Lewandowski, Brian G. Rector, Agnieszka Majer, Jarosław Raubic, Anna Skoracka

*corresponding author

Estimated costs of devices construction in USD.

Tunnel variant	Component**	Price/part [$]	Quantity	Cost [$]
‘Wind-transience’ tunnel	PMMA tube	22.00	1	22.00
	PVC tube	2.00	1	2.00
	Axial fan (Vents 100 VKO)	10.00	1	10.00
	Electrical wires	4.00	1	4.00
	PVC elbow connector	1.50	1	1.50
	Polyamide funnel	22.00	1	22.00
	Ø 12 mm aluminum tubes	0.50	26	13.00
	Ø 16 mm aluminum tubes	0.10	15	1.50
	Metal grille	3.00	1	3.00
	Wooden plank	1.50	1	1.50
	Brackets - Metal furniture legs 25 cm	4.00	2	8.00
	PVC Pipe Clamp	2.00	2	4.00
	Small accessories (e.g. screws)			0.50
	Power regulator (PAMEL PRD1)	32.00	1	32.00
	Programmer TS-EF1 BEMKO	8.00	1	8.00
TOTAL COST OF THE 'WIND-TRANSIENCE' TUNNEL				133.00
‘Vector-transience’ tunnel	Lego Mindstorms NXT 2.0	320.00	1	320.00
	PMMA tube	22.00	1	22.00
	Wooden plank	1.50	1	1.50
	PVC Pipe Clamp	2.00	2	4.00
	PVC tube	2.00	1	2.00
	Brackets - Metal furniture legs 15 cm	3.00	2	6.00
	Wool-covered ball	1.00	1	1.00
	Small accessories (e.g. screws, electrical wires)			0.50
TOTAL COST OF THE 'VECTOR-TRANSIENCE' TUNNEL				357.00
‘Departure’ tunnel	Axial fan (DP202A212, Sunon Taiwan)	10.00	1	10.00
	PMMA tube	4.00	1	4.00
	PVC tube	5.00	1	5.00
	Power regulator (PAMEL PRD1)	32.00	1	32.00
	Small accessories (e.g. screws, electrical wires)			0.50
TOTAL COST OF THE 'DEPARTURE' TUNNEL				51.50
Anemometer	Testo 405i anemometer	105.00	1	105.00
TOTAL COST OF THE ENTIRE DISPERSAL SYSTEM				646.50

**All materials are commonly available from local hardware stores or online retailers.
